# Pathway sensor-based functional genomics screening identifies modulators of neuronal activity

**DOI:** 10.1038/s41598-018-36008-9

**Published:** 2018-12-04

**Authors:** Alexander Herholt, Ben Brankatschk, Nirmal Kannaiyan, Sergi Papiol, Sven P. Wichert, Michael C. Wehr, Moritz J. Rossner

**Affiliations:** 1 Department of Psychiatry and Psychotherapy, University Hospital, LMU Munich, Munich, Germany; 2 Systasy Bioscience GmbH, Munich, Germany

## Abstract

Neuronal signal transduction shapes brain function and malfunction may cause mental disorders. Despite the existence of functional genomics screens for proliferation and toxicity, neuronal signalling has been difficult to address so far. To overcome this limitation, we developed a pooled screening assay which combines barcoded activity reporters with pooled genetic perturbation in a dual-expression adeno-associated virus (AAV) library. With this approach, termed pathScreener, we comprehensively dissect signalling pathways in postmitotic neurons. This overcomes several limitations of lentiviral-based screens. By applying first a barcoded and multiplexed reporter assay, termed cisProfiler, we identified the synaptic-activity responsive element (SARE) as top performance sensor of neuronal activity. Next, we targeted more than 4,400 genes and screened for modulatory effects on SARE activity in primary cortical neurons. We identified with high replicability many known genes involved in glutamatergic synapse-to-nucleus signalling of which a subset was validated in orthogonal assays. Several others have not yet been associated with the regulation of neuronal activity such as the hedgehog signalling members *Ptch2* and *Ift57*. This assay thus enhances the toolbox for analysing regulatory processes during neuronal signalling and may help identifying novel targets for brain disorders.

## Introduction

Functional genomics screens are the gold-standard for dissecting gene networks at the genome-wide level and contribute to target identification and mode-of-action studies in the biomedical field^1,2^. They are frequently used to identify genes with roles in proliferation, cell viability, viral infection, gene-drug interaction, and most recently cellular signalling^3–6^. The development has been mainly driven by cancer-related phenotypes (e.g. proliferation) in relatively easy accessible huge cell populations^7^. Unfortunately, these screening protocols are not suitable for the study of signalling in postmitotic cell types like primary neurons, as many disease-related neuronal phenotypes rely more on cell-to-cell communication (e.g. synaptic transmission) and the formation of complex morphologies and networks^8^. Existing protocols require high cell numbers per individual perturbation to gain sensitivity and robustness, but cell numbers are very limited for most primary postmitotic cell types^9^. The dissection of signalling pathways currently requires the dissociation of the cell population for cell sorting or droplet-based single cell analysis^10^. While this was successful in the case of immune cells and neuronal stem cells^4,11–14^, it is not a favourable procedure for mature neuronal networks and might generate preparation associated artefacts^15^. We reasoned that a protocol avoiding any trituration followed by cell sorting would greatly facilitate the screening process and enable pooled genetic screenings of so far inaccessible cell-types, such as primary neurons.

Neuronal activity-dependent signalling, e.g. modulating local protein synthesis and gene expression, is key to higher brain function and disturbed neuronal excitation and signalling has been associated with many brain diseases^16,17^. While descriptive methods such as proteomics and transcriptomics have delivered insight into the synaptic architecture and neuronal activity-dependent gene expression, respectively^8,18,19^, the empirical association of gene functions with a dedicated neuronal phenotype is a tedious endeavour and high-throughput techniques are not yet available. The activity state of neurons has long been visualized using reporter proteins controlled by activity-dependent promoters or enhancer elements^20–23^. We and others have demonstrated that signalling can be robustly measured by deep sequencing using pathway-specific reporters expressing short synthetic RNA barcode sequences^24,25^. Thus, we hypothesized that barcoded genetic sensors might provide the required sensitivity to perform comprehensive pooled screens for disturbed signalling in primary neuron cultures. In addition, the sensor approach would also circumvent the need for cell trituration and sorting.

As a proof-of-principle, we first identified a top neuronal activity sensor to build a sensor-coupled shRNA effector library targeting over 4,400 genes. Next, we applied this screening technology to silenced versus activated cortical neuron cultures to identify modulators of synapse-to-nucleus signalling.

## Results

### Pathway profiling identifies top performance neuronal activity sensor

**Figure 1:**
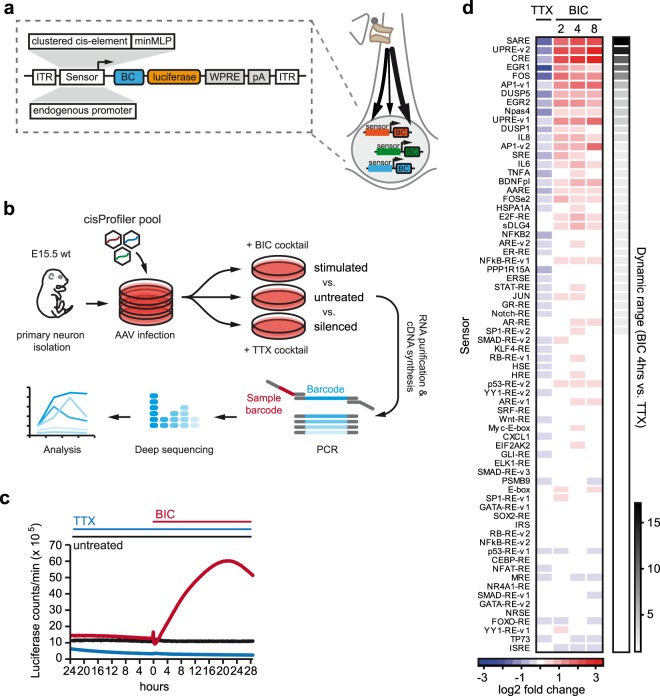
Pathway profiling in cortical neurons: Selection of a high-performance neuronal activity sensor. (**a**) Schematic map of the AAV cisProfiler vectors, used to simultaneously monitor multiple signalling events in neurons. See Supplementary Table S1 for details. (**b**) Workflow for the cisProfiler assay in primary cortical neurons. (**c**) Continuous live-cell recordings of the cisProfiler response in primary cortical neurons (DIV11–13) upon treatment with TTX cocktail (1 µM TTX, 100 µM APV) or BIC cocktail (50 µM BIC, 100 µM 4-AP, 100 µM glycine, 1 µM strychnine). Recording was performed in parallel to the assay shown in (**d**). (**d**) cisProfiler sensor responses at the indicated time points in primary cortical neurons as log2 fold-changes between treated (BIC, TTX) and untreated samples, measured by barcode sequencing. Sensors are ranked by the dynamic range between 4 hrs BIC cocktail stimulation and TTX cocktail treatment (timepoint used for pathScreener assay). Workflow as shown in (**b**). Treatment conditions as in (**c**). See Supplementary Table S2 for details. ITR, inverted terminal repeat; minMLP, minimal Major Late Promoter; BC, barcode; WPRE, Woodchock hepatitis virus posttranscriptional regulatory element; pA, poly-adenylation signal. BIC, bicuculline; TTX, tetrodotoxin; APV, (2R)-amino-5-phosphonovaleric acid; (2R)-amino-5-phosphonopentanoate; 4-AP, 4-Aminopyridine.

The sensitivity and robustness of a genetic screen is likely as dependent on the performance and dynamic range of the reporter as in cell-based compound screens^26^. Therefore, a panel of 70 cis-regulatory pathway reporters was generated to identify a sensor with excellent performance in a given cellular paradigm. The library consists of clustered transcription factor binding sites, enhancers, or short promoters coupled to a luciferase reporter and individually to unique RNA barcodes which we collectively term cisProfiler (Fig. 1a and Supplementary Table S1). The cisProfiler pool was packaged into AAV particles and used to infect postmitotic cortical cultures which were treated either with a cocktail including tetrodotoxin (TTX) to silence neuronal activity, or with a cocktail containing bicuculline (BIC) raising neuronal activity (Fig. 1b, see Methods for details).

Online monitoring of luciferase activity from the cisProfiler pool revealed a strong increase upon BIC stimulation, while TTX decreased the signal below baseline (Fig. 1c). To identify individual reporters mediating this response and in particular those displaying the largest dynamic range between an inactivated and activated state, we measured sensor activities by deep sequencing of the RNA barcodes (Fig. 1b) (see Methods for details). We identified a cluster of reporters which reacted to silencing and stimulation, containing regulatory elements of several prototypical neuronal-activity coupled genes such as *Fos* and *Egr1* among others (Fig. 1d, Supplementary Fig. S1, and Supplementary Table S2). Several sensors displayed kinetics with a sustained response, amplified response, or transient response, essentially reflecting corresponding categories described for endogenous genes induced by neuronal activity^27^.

The synaptic activity-responsive element (SARE) gave the largest dynamic range 4 hrs after BIC stimulation and outperformed classical neuronal activity reporters (Fig. 1d), in line with a previous report^28^. The SARE region is a conserved enhancer of the immediate-early gene *Arc*, consisting of binding sites for the transcription factors CREB, MEF2, and SRF/TCF, which can be found in promoters of multiple neuronal activity-dependent genes and is therefore likely integrating transcriptional responses elicited by different neuronal activity-dependent signaling pathways^29,30^. Given the high fold-change activation and well characterized structure of SARE, we decided to use this cis-regulatory element for developing the pathScreener assay. A cluster of four SARE repeats proximal to the 420 bp *Arc* core promoter (ArcMin) gave maximal dynamic range in luciferase reporter gene assays (Supplementary Fig. S2). This reporter is hereafter called E-SARE in accordance to the construct generated by Kawashima and colleagues and was subsequently used as final sensor element^28^.

### Construction of the AAV pathScreener library by sensor-effector coupling

**Figure 2:**
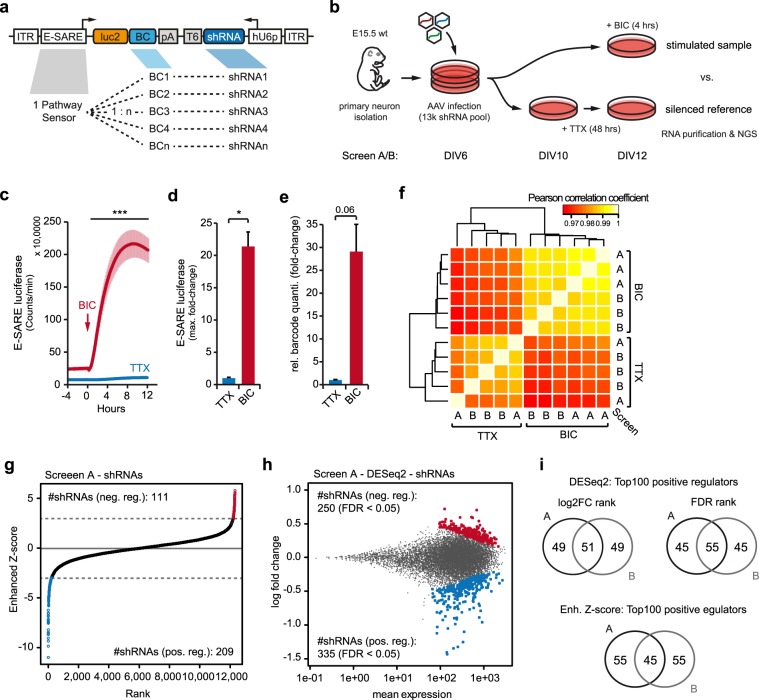
Pooled sensor-effector coupled interference screen in cortical neurons. (**a**) Schematic map of the pathScreener library vector. (**b**) Workflow for the pathScreener experiments in primary cortical neurons. (**c**) Continuous live-cell recordings of the pathScreener library response in primary cortical neurons (DIV12) upon treatment with TTX cocktail (1 µM TTX, 100 µM APV) or BIC cocktail (50 µM BIC, 100 µM 4-AP, 100 µM glycine, 1 µM strychnine). Recording was performed in parallel to screen A/B. n = 3, ±s.e.m. ***p < 0.001 (Two-way ANOVA). (**d**) Maximal E-SARE sensor induction during live-cell recording shown in (**c**). n = 3, ±s.e.m. *p < 0.05 (Two-tailed T-Test). (**e**) Relative barcode expression from pathScreener library in screen A after treatment with TTX cocktail for 48 hrs or BIC cocktail for 4 hrs. n = 2–3,±s.e.m. p = 0.06 (Two-tailed T-Test). (**f**) Pair-wise correlation of normalized read counts from biological replicates of screen A and B. Unsupervised hierarchical clustering. (**g**) Enhanced Z-score ranking for individual shRNAs from screen A. Enhanced Z-score threshold of ±3 is indicated by a dashed line. (**h**) DESeq2 analysis of screen A at shRNA level. MA-plot output comparing the sensor response for each shRNA within the library (log2 fold change: 4 hrs BIC cocktail vs. TTX cocktail; y axis) with the mean sensor expression (x axis). shRNAs with a significantly deregulated sensor expression (FDR < 0.1) are shown in red/blue. (**i**) Overlap between top 100 hit lists for positive regulators from screen A and B. Analysis by DESeq2 (top) or enhanced Z-score ranking (bottom). ITR, inverted terminal repeat; luc2, firefly luciferase; BC, barcode; pA, poly-adenylation signal; T6, terminator; shRNA, short hairpin RNA; hU6p, human U6 promoter. BIC, bicuculline; TTX, tetrodotoxin; APV, (2R)-amino-5-phosphonovaleric acid; (2R)-amino-5-phosphonopentanoate; 4-AP, 4-Aminopyridine. DIV, day-*in-vitro*. s.e.m., standard error of the mean.

Instead of a lentiviral screening vector, we used the AAV system as it has certain advantages. AAV particles have a natural tropism for neuronal cells and do not elicit cytotoxicity^31^. In addition, AAV-based systems have been extensively investigated for human gene therapy purposes and the availability of many natural as well as synthetic capsid proteins allows broad applications in many different tissues and cell types^32^. Furthermore, the AAV genome persists extrachromosomally which avoids effects on reporter performance depending on the genomic locus as with integrated lentiviruses. The AAV screening vector presented here was designed as a dual-expression vector containing the E-SARE reporter (=sensor component) and the shRNA cassette (=effector component) in opposing directions yielding the final sensor-effector coupled design (Fig. 2a). The E-SARE reporter is driving the expression of the Firefly luciferase (luc2) and a 35 bp barcode sequence (BC) in response to neuronal activity as in the cisProfiler assay (Fig. 1). The shRNA is constitutively expressed by a human U6 promoter, which performed better compared to neuronal promoters (Supplementary Fig. S3). We verified that the proximity of the shRNA expression cassette does not affect the E-SARE sensor performance (Supplementary Fig. S4). Run-through transcripts from the hU6 promoter due to insufficient termination have been a concern, as they would bias the barcode pool for sequencing if transcribed into cDNA^33^. A comparison of random primers with oligo(dT) primers for cDNA synthesis indicated that the oligo(dT) primers are superior in this set-up and we show that transcriptional run-through by RNA polymerase is not interfering with the assay (Supplementary Fig. S4).

We used a shRNA collection focused on 4,625 signalling pathway targets as template for library construction (see Methods for details). The shRNA cassette was amplified, extended by a short synthetic poly-adenylation signal (SpA) and a semi-random barcode sequence and cloned into the AAV vector containing the E-SARE reporter cassette (Supplementary Fig. S5). The proximity of barcode and shRNA within a window of less than 400 bp allowed assigning each shRNA with its barcode by deep sequencing (Supplementary Fig. S5) recovering ~97% of all target genes (see Methods for details).

### Pooled genetic interference screen in postmitotic cortical cultures

Within the library, the neuronal activity-dependent E-SARE sensor controls the expression of a multitude of unique RNA barcodes. Each barcode is linked to a specific shRNA expressed from the same AAV vector (Fig. 2a). Thereby, deep sequencing of the barcode pool during screening is sufficient to identify the corresponding shRNA and to measure the sensor activity simultaneously. We performed two screens with 2–3 replicates per condition. For each replicate sample, 10 or 5 million primary cortical neurons plated on 15 or 10 cm dishes, termed screen A and B respectively, were infected with the AAV library pool at an infection rate of ~60% at day-*in-vitro* (DIV) 6 avoiding multiple infections per cell (Supplementary Fig. S6). The maturation of the neuronal network was continued until DIV12 to allow formation of active synapses and spontaneous network activity^34,35^. Half of the cultures were subsequently silenced using a TTX cocktail to reduce the E-SARE activity to baseline level to quantify the relative abundance of each functional library vector. In the remaining cultures, network activity was stimulated by a BIC cocktail in order to reveal genes which participate in neuronal activity-dependent signalling (see Methods for details). From each sample, total RNA was purified, and barcode pools were prepared for deep sequencing. The effect of each gene knockdown on E-SARE activity was determined by comparing all barcodes of the stimulated samples with each other after normalization to the silenced reference samples (Fig. 2b). It was expected that a knockdown of a positive regulator of activity-dependent neuronal signalling will decrease the relative E-SARE activity, whereas a knockdown of a negative regulator will increase the relative E-SARE activity. The induction of the E-SARE activity upon stimulation of synaptic activity by BIC was monitored in sister cultures by live cell online luciferase recordings (21 fold-change, BIC vs TTX) (Fig. 2c,d). The induction of barcode expression from the E-SARE reporter in the actual screening samples was also controlled using relative quantification by qRT-PCR of all barcodes in the library (29 fold-change, BIC vs TTX) (Fig. 2e). Normalized read counts of replicate samples from two screens (‘A’ with 10 million and ‘B’ with 5 million cells per sample) highly correlated within conditions, highlighting the robustness of the screening technology (Pearson correlation coefficient ρ > 0.985 BIC; ρ > 0.972 TTX) (Fig. 2f). These results also indicate that the screening coverage may be further decreased below 250 cells per individual shRNA, as applied in screen B, which is far below the suggested coverage needed for Lentiviral screens with >10^3^ cells per construct^9^.

We used an enhanced Z-score ranking (ZS) and the Bioconductor DESeq2 package as two independent analysis methods for hit nomination (Table 1, Supplementary Table S3)^4,36^. Rankings from the two analysis methods were highly correlated (Spearman rank coefficient *rho* > 0.972, Screen A/B; Supplementary Fig. S7) and we observed a considerable number of shared hits among the top 100 positive regulators (54/100, p < 10^−10^, hypergeometric test; Supplementary Fig. S7). In both approaches, more shRNAs scored for positive regulators of neuronal activity, thus reducing the E-SARE activity (Screen A ZS: 209 vs. 111 shRNAs, cutoff at enhanced ZS ± 3; DESeq2: 335 vs. 250 shRNAs, cut-off at FDR < 0.05) (Fig. 2g,h, and Supplementary Fig. S7). Overall, hit gene rankings from screen A and B are correlated (Spearman rank coefficient *rho* = 0.567; Supplementary Fig. S7), with a substantial overlap for the top 100 positive regulators between the replicate screens (45/100 for enh. ZS, 55/100 for DESeq2, p < 10^−10^, hypergeometric test; Fig. 2i). The assay in the current setup was less sensitive for negative regulators likely due to the strong and nearly saturating stimulation by the BIC cocktail, which was reflected by less primary negative regulator hits and a smaller overlap between individual screens (15/100 for enh. ZS, p < 10^−8^, hypergeometric test; Fig. 2g,h, and Supplementary Fig. S7).

**Table 1:** Top 25 positive regulator hits.

Gene symbol	Refseq ID	Enh. ZS	DESeq2		
			log2FC	p-value	p-adj
*Vegfa*	NM_001025250	−11.01	−0.913	2.77E-10	NA
*Adcy3*	NM_138305	−9.87	−1.430	9.35E-23	1.67E-19
*Il2rb*	NM_008368	−9.34	−1.399	1.68E-22	2.58E-19
*Igfbp6*	NM_008344	−9.07	−1.119	1.22E-13	8.21E-11
*Camk2d*	NM_001025439	−8.71	−1.343	3.29E-21	4.42E-18
Cyp3a13*	NM_007819	−8.59	−0.707	1.36E-06	NA
*Cflar*	NM_207653	−8.46	−1.028	1.02E-11	4.57E-09
Fgf18*	NM_008005	−8.23	−0.413	2.57E-08	5.73E-06
Cox15*	NM_144874	−7.58	−1.320	2.09E-24	7.49E-21
*Dnajb1*	NM_018808	−7.48	−0.653	9.79E-06	NA
*Hr*	NM_021877	−7.45	−0.219	3.00E-02	2.09E-01
Ptch2*	NM_008958	−7.36	−0.808	9.44E-08	1.75E-05
Adh1*	NM_007409	−7.25	−1.048	8.12E-13	4.59E-10
*Il27*	NM_145636	−7.17	−0.601	4.01E-05	NA
Araf*	NM_009703	−6.88	−0.521	9.35E-06	NA
*Dpysl2*	NM_009955	−6.79	−0.818	6.52E-08	1.30E-05
*Mapk14*	NM_011951	−6.57	−1.249	5.51E-28	3.00E-24
Krt18*	NM_010664	−6.55	−0.921	4.24E-10	1.52E-07
Dot1l*	NM_199322	−6.23	−0.870	4.11E-09	1.13E-06
Pigm*	NM_026234	−5.81	−0.795	8.89E-08	1.70E-05
Xcr1*	NM_011798	−5.73	−1.096	5.85E-23	1.26E-19
*Cyp19a1*	NM_007810	−5.71	−0.573	1.38E-04	6.49E-03
Ift57*	NM_028680	−5.67	−0.981	5.84E-14	4.47E-11
Apbb2*	NM_009686	−5.42	−0.416	3.69E-03	NA
*Ppp5c*	NM_011155	−5.41	−0.551	2.52E-04	NA

Top 25 Hits ranked by enhanced Z-score (Enh. ZS) along with corresponding log2 fold change values (log2FC) and unadjusted (Wald statistics) and adjusted p-values (Benjamini-Hochberg) from the DESeq2 analysis. Gene Symbols marked with * have not yet been associated with neuronal signalling according to a detailed PubMed search (see below). NA, adjusted p-value could not be calculated. Continued in Supplementary Table S3.

### pathScreener identifies genes involved in neuronal signalling and brain disorders

**Figure 3:**
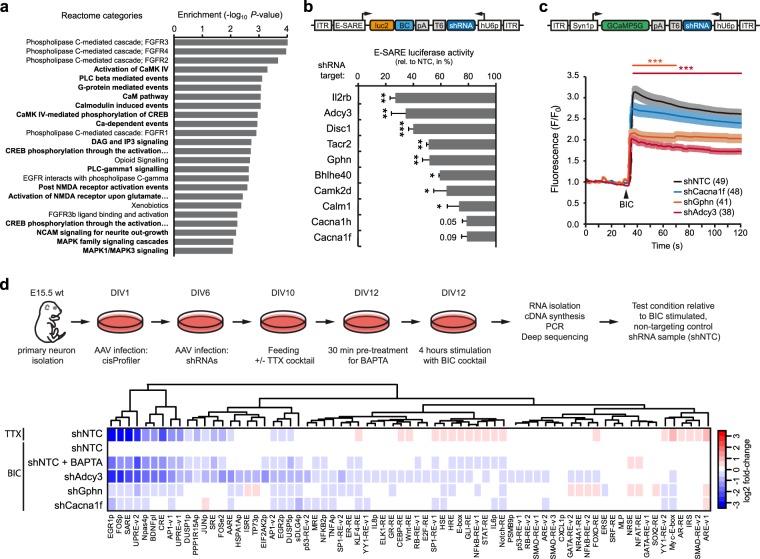
Orthogonal validation of selected primary hits. (**a**) Reactome pathway analysis of top 150 positive regulator hits from screen A based on Z-score ranking. Notably, multiple terms related to synapse-to-nucleus signalling are among the top categories (bold). (**b**) Individual validation of selected hit shRNAs in E-SARE luciferase assay. All tested shRNAs reduced E-SARE activity relative to the non-targeting control (NTC). Same cell culture paradigm as in screens A/B (Fig. 2b). n = 3, ±s.d. ***p < 0.001; ***p < 0.01; *p < 0.05 (Two-tailed T-Test). (**c**) Effect of *Adcy3*, *Gphn*, and *Cacna1f* knockdown on calcium dynamics after stimulation with BIC cocktail, measured using GCaMP5G. Number of cells in parenthesis. Normalized mean intensity values ± s.e.m. ***p < 0.001 (Two-way ANOVA). (**d**) Effect of *Adcy3*, *Gphn*, and *Cacna1f* knockdown on multiple signalling pathways measured using the cisProfiler library. Cortical cultures infected with hit shRNAs were stimulated with BIC cocktail (BIC) for 4 hr. For comparison, the heatmap also shows profiles of silences cultures (TTX) and cultures were calcium signalling was abolished (BAPTA). Responses were normalized to non-targeting control (shNTC) samples stimulated with the BIC cocktail. See Supplementary Table S6 for details. BIC, bicuculline; TTX, tetrodotoxin; BAPTA, 1,2-bis(o-aminophenoxy)ethane-N,N,N′,N′-tetraacetic acid; s.e.m., standard error of the mean; s.d., standard deviation.

In order to systematically interpret the screening results, we tested for pathway enrichment in manually curated gene sets from Reactome and KEGG databases^37,38^. For the top-ranked positive regulators, the Reactome categories “Ca-dependent events”, “CaMK IV-mediated phosphorylation of CREB”, and “Activation of NMDA receptor upon glutamate binding and postsynaptic events” (all p < 0.003 and false discovery rate [FDR] q val < 0.05; Fig. 3a, and Supplementary Table S4), as well as the KEGG pathway categories “Long-term potentiation”, “Neurotrophin signaling”, and “Calcium signaling” (all p < 0.05, hypergeometric test; Supplementary Table S5) were identified amongst others related to synapse-to-nucleus signalling.

Based on a cut-off at an enhanced ZS of −2, we next selected ten high, mid, and low-ranking genes from the list of positive regulators and cloned the corresponding shRNA of each candidate individually into the dual-expression vector (Fig. 3b). For each construct, AAVs were generated and neurons were infected on DIV6 followed by cell lysis and luciferase assays on DIV12 as for the primary screen. Among these candidate shRNAs, eight significantly reduced the SARE-driven luciferase activity upon BIC stimulation compared to the non-targeting control (NTC) vector (all p < 0.05). Knockdown of the two L-type Ca-channel units CACNA1F and CACNA1H revealed lower levels of reductions with effects close to significance (p = 0.05 and 0.09, respectively) (Fig. 3b).

From the above validated candidates, we further focused on adenylate cyclase 3 (*Adcy3*), gephyrin (*Gphn*) and the alpha 1F subunit of the voltage-dependent L-Type calcium channel (*Cacna1f*) and cloned corresponding shRNAs into a dual-expression AAV backbone allowing to assess Ca^2+^-imaging via the GCaMP5G reporter as an orthogonal validation assay (Fig. 3c). Knockdown of *Adcy3* and *Gphn* reduced BIC-induced Ca^2+^ release compared to a non-targeting control (NTC) vector significantly (p < 0.001). Again, the shRNA directed against the L-Type calcium channel *Cacna1f* revealed the most moderate effect (Fig. 3b) which was congruent with the observed knockdown effects assessed by qRT-PCR (Supplementary Fig. 8). Finally, we re-applied the cisProfiler assay to monitor the consequence of *Adcy3*, *Gphn* and *Cacna1f* knockdowns compared to Ca^2+^-depletion mediated by BAPTA in neurons (Supplementary Table S6). Unsupervised clustering of all pathway reporter responses revealed graded effects of all three candidates on multiple neuronal activity sensors (e.g. EGR1, FOS, Npas4, BDNFpI) with knockdown of *Adcy3* showing the most pronounced suppression of reporter activities followed by *Gphn* and *Cacna1f*, in line with the GCaMP results (Fig. 3d).

**Figure 4:**
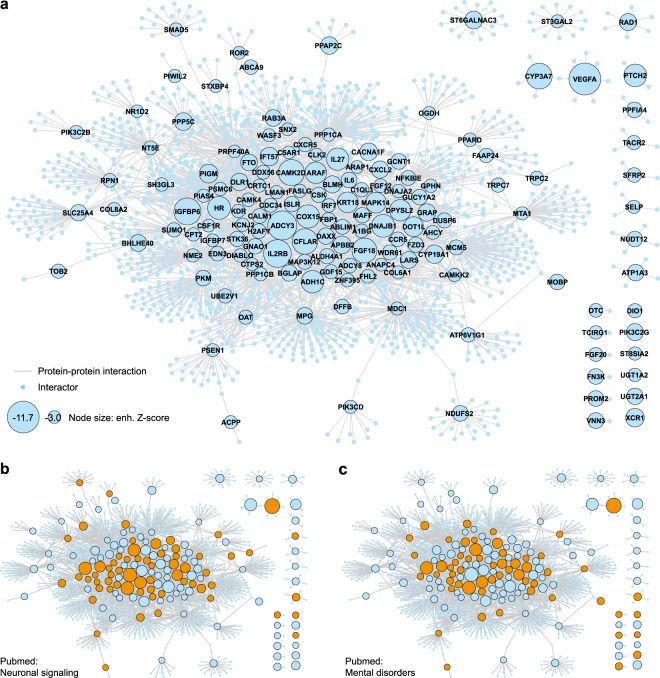
Network visualization of top primary hits. (**a**) Network visualization of top positive regulator hits from screen A identifies one main cluster connected by multiple protein-protein interactions as well as several unconnected hits. Gene names are shown as node titles. Node size reflects enhanced Z-score values. Edges represent known protein-protein interactions (Cytoscape/Bisogenet) and interactions with one additional interactor are shown (see Methods for details). (**a**,**c**) Network as in a, highlighting genes (orange nodes) that belong to Reactome pathway categories related to synapse-to-nucleus signalling (**b**, refers to Fig. 3a, see Supplementary Table S4) or those referenced in PubMed with MeSH terms related to neuronal signalling (see Methods for details on PubMed query) (**c**).

Finally, we visualized the topology of the top-ranked positive regulators in an extended protein-protein interaction network, where most candidates (106/130, 82%) were connected in a tight cluster indicating functional relationships (Fig. 4a). A high proportion of these hits are associated in the literature with MeSH terms related to neuronal signalling such as ‘synaptic transmission’, ‘neuronal plasticity’, or ‘long-term potentiation’ (59/130, 45%; Fig. 4b) or mental disorders like “Schizophrenia Spectrum and Other Psychotic Disorders” (53/130, 40%; Fig. 4c) (see Methods for all details of the PubMed query). Nonetheless, for a reasonable number of candidates no literature association was found. These include for example the hedgehog signalling components *Ptch2* and *Ift57*, as well as multiple chemokine signalling members (*Cxcr5*, *Ccr5*, *Cxcl2*, *Xcr1*) which ranked among the top hits (Table 1, Supplementary Table S3).

We conclude from these analyses that the pathScreener assay can identify known and novel players of neuronal signalling with high confidence and provides an empirical tool to characterize components of complex signalling pathways.

## Discussion

The technical challenges of functional genomics screens in primary neurons following standard, abundance-based protocols are indicated by a recent small scale CRISPR/Cas9 screen where small effect sizes and low replicate correlations were reported^39^. In contrast, we could show that the design of sensor-effector coupling gives sensitive and robust readouts, leading to a high degree of reproducibility across screens and avoiding any cell sorting induced bias^15^. Nonetheless, Kramer *et al*. identified so far unknown modulators of amyotrophic lateral sclerosis-related neurodegeneration, highlighting the importance of genomics screens in neuronal systems. With the increasing interest in primary cell cultures for functional genomics screens, the aspects of cell heterogeneity as well as network- versus cell-intrinsic effects are new topics to the field and require consideration during data interpretation. This is of importance for neuronal cultures where many cells (and different cell types, see below) interact and modulate each other. A pooled screen with a neuronal network is always limited to cell-intrinsic effects of the genetic interference, as only a fraction of cells will have a certain gene-specific phenotype. However, during orthogonal validation it is possible to discriminate between cell-intrinsic and network level effects by infecting at low versus high titers and analysing shRNA and non-shRNA infected neurons with control conditions. Cell heterogeneity leads to screening results that are rather generic across all cell types within a culture. This also applies to the primary mouse cultures used here, even though most cells are excitatory neurons. To allow a more precise cell type focused analysis, the pathScreener approach could be combined with a genetic label such as Cre-recombinase or FACS purified cell populations.

With over 4,400 targets included within our library, it allowed us to screen an unprecedented number of genes in primary cortical neurons and to retrieve many known and unknown modulators of neuronal activity. Notably, we successfully screened at much lower cell numbers per shRNA than the standard for pooled screens (250 vs >1,000 cells per shRNA/sgRNA). Despite this low cell to shRNA ratio, replicate correlations were outperforming those of a FACS-based signalling screen in dendritic cells and those of the above mentioned toxicity screen in neurons^4,39^.

The reproducibility and depth of the network deconvolution are likely to be further increased using CRISPR/Cas9 libraries. Side-by-side comparisons of pooled RNAi and CRISPR/Cas9 screens argue for higher specificity and larger effect sizes in the case of CRISPR/Cas9^40,41^. As a trade-off, CRISPR/Cas9 screens essentially dependent on the co-expression of a Cas9 effector-mediating protein (e.g. in neurons) which is not needed for RNAi. Nevertheless, RNAi and CRISPR/Cas9 screens should be considered as complementary approaches, as both seem to have advantages in identifying distinct classes of genes in certain screening paradigms^42^. In particular, if functional genomics screens are performed towards drug target identification, a gene knockdown might more likely phenocopy a drug action than a knockout^5^.

This proof-of-concept screen did already identify a reasonable number of primary hits without published association with neuronal function or contributions to mental disorders. Among the top hits from both screen replicates, we for example found *Ptch2* and *Ift57* which are members of hedgehog signalling and the intraflagellar transport machinery^43^. These biological processes are well known for their role in primary cilium function during nervous system development and axon outgrowth. Still little is known about the role of hedgehog signalling in synaptic plasticity^44,45^. Our screening design was focusing on a time-window where axonal specification and primary dendrite outgrowth is completed, and synaptogenesis and spine maturation takes place^46^. The fact that *Ptch2* and *Ift57* appear as top hits provide new evidence that hedgehog signalling might also regulate synaptic plasticity and neuronal activity. This finding definitively requires thorough follow up validations to be substantiated. Thus, among all unreported modulators there might be putative targets for drug discovery to modulate neuronal activity. Follow-up studies are needed to investigate such candidates and to individually validate their functions.

Taken together, the pathScreener assay allows to down-scale cell culture sizes, making genome-wide screens in primary neurons truly feasible. The general strategy presented, i.e. first identifying a top-performance sensor from the cisProfiler library, followed by a pathScreener assay to dissect upstream signalling cascades, has the potential to be modified towards diverse applications, such as expanding the sensor repertoire for different specificities and/or cell types towards human cellular disease models.

## Methods

All methods were carried out in accordance with institutional guidelines and regulations including animal experiments (breedings of C57bl6 mice for generation of primary neurons) approved by the Regierungspräsidium Oberbayern, ROB Munich, Germany under the license Az: 55.2-1-54-2532-179-2016.

### Chemicals

Tetrodotoxin citrate (TTX), (−)-Bicuculline methiodide (BIC), 4-Aminopyridine (4-AP), Strychnine hydrochloride, Glycine (all Abcam), (2 R)-amino-5-phosphonovaleric acid; (2 R)-amino-5-phosphonopentanoate (D-APV) (R&D Systems), Phorbol-12-myristat-13-acetat (PMA), 1,2-bis(o-aminophenoxy)ethane-N,N,N′,N′-tetraacetic acid) (BAPTA) (Sigma).

### Cell culture

Primary neurons were isolated from cortices of E15.5 C57BL6/N mice from in-house facilities. Cortices were dissociated by treatment with Papain suspension (Worthington) and gentle trituration. Cell plating was done in Neurobasal medium supplemented with 2% B27, GlutaMax and 5% FBS (all Gibco), on poly-L-lysine (MW 70,000–150,000; Sigma) coated plastic tissue culture dishes (BD Falcon) at a cell density of 550–650 cells/mm^2^. On DIV1, plating medium was replaced by Neurobasal medium supplemented with 2% B27 and GlutaMax. Cultures were fed for the first time on DIV5/6 and then every 3–4 days.

SH-SY5Y cells (ATCC) were cultured in DMEM (high glucose) supplemented with 10% FBS and GlutaMax (all Gibco). PC12 Tet-Off cells (Clontech, termed PC12 cells for simplicity) were cultured in DMEM (low glucose) supplemented with 10% FBS, GlutaMax and penicillin/streptomycin (all Gibco). HEK293FT cells (ATCC) were cultured in DMEM (high glucose) supplemented with 10% FBS and GlutaMax (all Gibco).

All cells were grown in a humidified incubator (37 °C, 5% CO_2_).

### AAV production

The production of AAV particles was done following a previously published protocol with minor modifications^47^. In brief, HEK293FT cells were co-transfected with the AAV vector (or library) and the pFdelta6 helper plasmid as well as an equimolar mix of the capsid plasmids pH21 and pRV1 (serotype 1 and 2, respectively) using polyethylenimin transfection reagent (Polyscience). Cells were lysed by freeze-thaw cycles to release the AAV particles and treated with Benzonase (Sigma) to digest genomic DNA. The lysate was cleared by centrifugation and filtration through a 0.45 µm syringe filter (Millipore), and finally concentrated using an Amicon Ultra-15 centrifugal filter unit (100 kDa membrane cut-off; Millipore) including a PBS wash.

The AAV titer was determined by absolute quantification of DNase-resistant AAV genomic copies (GC) using quantitative PCR. Therefore, an AAV stock sample was first treated with TURBO DNase (Invitrogen). After inactivation of the DNase, the AAV capsid was digested by proteinase K (Invitrogen) and AAV genomes were isolated by spin column-based purification (Macherey&Nagel). shRNA containing AAVs were quantified with primers against the hU6 promoter. All other AAVs were quantified with primers against the WPRE sequence.

In order to estimate the ratio of infectious AAV particles to genomic copies, primary neurons were infected with a serial dilution of a GFP expressing AAV. GFP positive and total (DAPI positive) cells were counted to calculate the infection rate for a given multiplicity of infection (MOI) (Supplementary Fig. S6).

### Cloning of the cisProfiler library

The AAV cisProfiler cloning vector contains AAV2 ITRs flanking a Gateway recombination cassette (Invitrogen) followed by the *firefly* luciferase ORF (luc2, Promega), the WPRE sequence and a BGH polyA signal. MultiSite Gateway recombination (Invitrogen) was used to generate the cisProfiler sensor vectors. These vectors exist in two configurations of the MultiSite Gateway cassette, depending whether the sensor is an endogenous promoter or a clustered cis-element:

Endogenous promoter: attB1_stuffer_attB4r_promoter_attB3r_barcode_attB2

Clustered cis-element (incl. enhancer): attB1_cis-element_attB4r_MLP_attB3r-barcode-attB2

cisProfiler sensor sequences were PCR amplified from a genomic DNA template or synthesized (Genscript) and cloned into a pDONR shuttle vector (Invitrogen) (see Supplementary Table S1 for details). The remaining pDONR vectors for the MultiSite Gateway recombination contain a stuffer sequence, or the adenovirus major late promoter (MLP), or a 49mer barcode library. The barcode design has been previously described^24^. After MultiSite Gateway recombination, each AAV cisProfiler vector has been verified individually by Sanger sequencing and three distinct barcode replicates were selected per sensor and included into the cisProfiler library with few exceptions (Supplementary Table S1). Vectors with only the MLP, but no sensor, were included as internal calibrators. For the cisProfiler assays, the library has been packaged into AAV particles as a pool.

### cisProfiler assay

The cisProfiler assay was conducted with primary cortical neurons. Per assay, 5 ∗ 10^5^ cells were plated and infected with the AAV cisProfiler library on DIV5 at a MOI of 2,500. A duplicate sample was silenced with 1 µM TTX and 100 µM APV at DIV12. On DIV14, a duplicate sample was harvested as an untreated reference. The remaining samples were stimulated with 50 µM bicuculline, 100 µM 4-AP, 100 µM strychnine and 100 µM glycine (BIC cocktail) for 2, 4, and 8 hours in duplicates. All samples were finally harvested in QIAzol reagent (Qiagen) and the total RNA was purified using the Direct-zol RNA MiniPrep kit (Zymo). The purified RNA was treated with TURBO DNase (Invitrogen) to digest residual AAV genomes and cleaned up by a second spin-column purification using the Direct-zol RNA MiniPrep kit according to the manufacturer’s instructions.

Total RNA was reverse transcribed into cDNA using the SuperScript III RT (Invitrogen) and random nonamer primers. The barcode sequences were amplified by PCR with barcode flanking primers (Dec_fwd and Dec_rev). In a second round of PCR, the Ion Torrent adapter Ion-A and a 12 bp sample barcode was added to the 5′ end and the Ion Torrent adapter Ion-P1 to the 3′ end. All PCRs were performed with HotStar Taq plus DNA polymerase (Qiagen). The PCR products for each sample were verified by agarose gel-electrophoresis, pooled and purified using the NucleoSpin Gel and PCR Clean-up kit (Macherey&Nagel).

The pooled barcode sample was sequenced on an Ion Torrent PGM sequencer (Life Technologies) according to the manufacturer’s protocols. Processing of the raw data was done using custom shell and R scripts. Raw reads were first split into individual samples using the 12 bp sample barcode. Next, reads were aligned to a reference barcode library using a local BLAST. For analysis, read counts were first normalized to total reads per sample and then to the read counts of the MLP-only control vectors. The normalized read counts of the barcode replicates per sensor were averaged first and then the average of the sample replicates was calculated. Finally, data was presented as log2-transformed fold changes relative to the reference sample.

Nonparametric Mann–Whitney test was applied to compare TTX and BIC groups at the different time points analysed in these experiments (T2, T4, T8) using IBM SPSS Statistics v22 (IBM SPSS, Statistics for Windows, Version 220. IBM Corp.: IBM Corp: Armonk, NY, USA, 2013). Multiple testing correction was carried out using false discovery rate (FDR). The p.adjust() function in R 3.2.1 software (R Core Team, R: A Language and Environment for Statistical Computing. R Foundation for Statistical Computing: Vienna, Austria, 2015) was used for this purpose. See Supplementary Table S2 for the cisProfiler statistics.

### Live-cell luciferase assay

Live-cell luciferase recordings were performed as quality controls during cisProfiler- and pathScreener assays^48^. 5 ∗ 10^5^ primary cortical neurons were seeded in 3.5 cm tissue culture dishes and cultured in a LumiCycle 32 apparatus, inside a non-humidified incubator (37 °C, 5% CO_2_). The cell culture medium was supplemented with D-luciferin (Promega). Cultures were treated according to the experimental paradigm of the cisProfiler- or pathScreener assay. Two-way ANOVA was applied for statistical analysis (GraphPad Prism).

### Multiplate luciferase reporter gene assay

For multiwell-plate assays, cells were seeded into 96-well plates and either transfected (for cell lines) or infected (for primary neurons). Cells were transfected using Lipofectamine2000 (Invitrogen) according to the manufacturer’s instructions or infection by an E-SARE-luciferase containing AAV. Infection was done with 500–1,000 AAV GCs per cell.

Validation of individual shRNAs was done with primary neurons in 24-well plates. Neurons were infected with AAV pathScreener vectors either expressing a shRNA or a non-targeting control RNA. Cultures were treated the same way as the screening samples (BIC cocktail vs. TTX cocktail). At the end of the assay, cells were lysed using Passive lysis buffer (Promega). The luciferase activity was measured by a Mithras LB 940 Microplate Reader (Berthold Technologies)^26^. Two-sided Student’s T-Test was applied for statistical analysis.

### pathScreener vector construction

The pathScreener library cloning vector contains AAV2 ITRs, flanking the sensor and effector cassettes. The sensor cassette comprises of a multiple cloning site (MCS) for sensor insertion, followed by the *firefly* luciferase ORF (luc2, Promega), the barcode cassette and a synthetic poly-adenylation signal (SpA)^49^. The effector cassette, which is in the reverse orientation, harbours the human U6 (hU6) promoter, a stuffer sequence for shRNA insertion, and a T6 terminator sequence.

The E-SARE sensor was cloned into the MCS using KpnI and HindIII sites. Test shRNAs were cloned downstream of the hU6 promoter using AgeI and EcoRI sites. To examine interference between the sensor and the effector cassette, sensor or effector deletion vectors were cloned (Supplementary Fig. S4). Therefore, either the E-SARE sensor or the hU6p-shRNA cassette was excised with KpnI/HindIII or ClaI/EcoRI, respectively, and cut sites were blunted using T4 DNA Polymerase and re-ligated.

### pathScreener library construction

For construction of the pathScreener library, we used the DECIPHER shRNA Mouse Module 1 library (Cellecta) as a template. This library has an optimized shRNA design and is focused on 4,625 signalling pathway targets. It contains 5–6 shRNAs per target. The insert for library cloning was prepared by two rounds of PCR. In the first PCR, the hU6p-shRNA cassette from the DECIPHER library was fused to an oligo containing the synthetic poly-A signal (library_SpA oligo) and amplified by flanking primers (library_Dec_rev/library_hU6_fwd). Next, an oligo with a 35 bp semi-random barcode (library_BC35 oligo) was fused by PCR to the purified PCR product of PCR#1 and amplified with flanking primers (library_hU6_fwd/library_BC_rev). The resulting insert was cut with BamHI and ClaI and spin-column purified.

The pathScreener vector, containing the E-SARE sensor, was cut with the same restriction-enzyme pair and the linearized vector was first purified by agarose gel-extraction using the NucleoSpin Gel and PCR Clean-up kit (Macherey&Nagel). In addition, the vector was purified by phenol/chloroform extraction and ethanol precipitation.

Library insert and linearized pathScreener vector were ligated using T4-ligase. Due to the short 2 bp overhang at the ClaI site, ligation was slightly modified according to a published protocol for enhanced 2 bp overhang cloning^50^. In brief, the vector/insert mix was heated to 55 °C and subsequently frozen at −20 °C before overnight ligation at 16 °C.

### shRNA-barcode assignment by deep sequencing

In order to assign the barcode sequence to the shRNA, the region encoding the barcode and the shRNA was amplified by PCR using primers with Ion Torrent sequencing adapters (PGM_A_IXcode3_AFA_s / PGM_trP1_hU6_as). The PCR product has a size of 345 bp and was sequenced on an Ion Torrent PGM sequencer using the 318 chip and the Ion PGM Template OT2 400 Kit (Life Technologies) for 400 bp read length. Sequencing was done according to the manufacturer’s protocols. The analysis and assignment of barcode sequences to shRNAs was finally done using custom R scripts. See Supplementary Table S7 for shRNA sequence information.

### Interference screens with pathScreener library

#### Cell culture

Primary cortical neurons from 16 embryos were pooled per screen. Per sample, 10 or 5 million cells were seeded onto PLL-coated 15 cm or 10 cm tissue culture dishes, respectively. In parallel, PLL-coated 3.5 cm dishes were seeded with 5*10^5^ cells to monitor the treatments using the Lumicycler. 2–3 replicate cultures were prepared per treatment condition. On DIV6, cultures were infected with the AAV pathScreener library at MOI 1,000. On DIV10, half of the cultures were treated with 1 μM TTX and 100 μM APV (TTX cocktail) to silence neuronal activity. The cultures for live-cell luciferase recordings were in addition supplemented with the firefly luciferase substrate D-luciferin (Promega) and the recording using the Lumicycler was started. On DIV12, the cultures which have not been silenced were stimulated with a cocktail containing 50 μM BIC, 100 μM 4-AP, 100 μM glycine, 1 μM strychnine (BIC cocktail) for 4 hours.

#### Harvest

All cultures were lysed after a PBS wash with QIAzol cell lysis reagent (Qiagen) (5 ml for 15 cm dishes, 2.5 ml for 10 cm dishes). Cell lysate was scraped from the dishes and transferred into a 15 ml tube. The lysate was kept at −80 °C until RNA isolation.

#### Total RNA isolation

Cell debris in the lysate was pelleted by centrifugation at 4,000 rpm for 5 minutes. The supernatant was transferred into a new 15 ml tube and the total RNA was isolated using the Direct-zol RNA MiniPrep kit (Zymo) according to the manufacturer’s instructions with the following modifications. The lysate from 10 million cells was split onto 2 RNA purification spin-columns in order to not exceed the RNA binding capacity of a column. Elution was done in 50 μl RNase-free H2O per column and the two eluates from 10 million cells were pooled afterwards.

In order to digest traces of co-isolated AAV genomes, total RNA was treated with TURBO DNase (Invitrogen) for 30 minutes at 37 °C and subsequently cleaned-up using the Direct-zol RNA MiniPrep Kit (Zymo) for purification. One column was used per sample. Elution was in 25 μl RNase-free H_2_O.

#### cDNA synthesis

The first-strand cDNA synthesis was done using the SuperScript III reverse transcriptase (Invitrogen). The entire total RNA was reverse transcribed in multiple 20 μl reactions containing 5 μg total RNA each and using oligo(dT) primer. The reaction protocol was as followed:

**Table:** 

Total RNA	5 µg
Oligo(dT) primer (50 μM)	1 µl
dNTPs (10 mM each)	1 µl
H_2_O	up to 13 µl

5 minutes at 65 °C, followed by 1 minute on ice. Then add per reaction:

**Table:** 

5x First-strand reaction buffer	4 µl
DTT (0.1 M)	1 µl
H_2_O	1 µl
SuperScript III RT	1 µl

Incubate first at 50 °C for 30 minutes, followed by 15 minutes at 70 °C.

#### Barcode quantification by qRT-PCR

In order to validate the sensor induction during the screen, RNA barcode expression was quantified relative to Rpl13a expression. Primer pairs were qDec1.2/qDec2.2 for the barcode and qRT-PCR primer for Rpl13a. Analysis was done using the Qiagen Rotor-Gene Software with the ΔΔCt-method for relative quantification.

**Table:** 

2x RotorGene SYBRgreen PCR Master Mix	5 µl
Fwd primer (10 μM)	1 µl
Rev primer (10 μM)	1 µl

Default qRT-PCR cycling parameters.

#### Dec PCR

The ‘Dec PCR’ amplifies the barcode from the cDNA sample. Prior to the ‘Dec PCR’, the entire cDNA was purified using the PCR clean-up kit (Macherey&Nagel) and eluted with 20 μl elution buffer. Per sample 100 μl reactions were prepared, split into 2 × 50 μl reactions for PCR and pooled again afterwards.

**Table:** 

cDNA (purified)	10 µl
qDec1.2 fwd primer (10 μM)	1.25 µl
qDec2.2 rev primer (10 μM)	1.25 µl
H2O	37.5 µl
NEBNext 2x PCR MasterMix	50 µl

PCR parameters: 98 °C 30 sec, 98 °C 10 sec, 59 °C 30 sec, 72 °C 30 sec (20 cycles).

The PCR product was confirmed by 2% agarose gel-electrophoresis.

#### Code PCR

The ‘Code PCR’ fuses sample specific 12 bp code sequences to the ‘Dec PCR’ product in order to pool samples for next-generation sequencing. The forward code primer contains the Ion-A adapter sequence required for Ion Torrent sequencing and the 12 bp code sequence. The reverse primer contains the Ion-P1 adapter sequence required for Ion Torrent sequencing. Code PCR reaction per screen sample:

**Table:** 

Dec PCR product (pre-diluted 1:10)	5 µl
Code fwd primer (10 μM)	0.625 µl
Code2.2 rev primer (10 μM)	0.625 µl
H2O	18.75 µl
NEBNext 2x PCR MasterMix	25 µl

PCR parameters: 98 °C 30 sec, 98 °C 10 sec, 58 °C 30 sec, 72 C 30 sec (10 cycles).

The PCR product was confirmed by 2% agarose gel-electrophoresis.

20–40 μl per sample were pooled subsequently and purified using the NucleoSpin Gel and PCR Clean‐up kit (Macherey&Nagel).

#### Next-generation sequencing of barcodes

Barcode libraries were sequenced on an Ion Torrent Proton sequencer using the PI chip (Life Technologies). All template preparations and enrichments were done according to the manufacturer’s protocols for the Ion PI Template OT2 200 v3 kit (Life Technologies). Sequencing was done according to the manufacturer’s protocols for the Ion PI Sequencing 200 v3 kit. One PI chip delivered on average over 100 million raw reads.

Processing of the raw data was done using custom shell and R scripts. First, raw reads were split into individual samples using the 12 bp code and subsequently mapped to a reference barcode library using a local BLAST. Thereby, reads were counted and assigned to shRNAs and gene targets. Next, read counts were normalized to total read numbers per sample. If multiple barcodes are assigned to the same shRNA, corresponding read counts were summed. To control the correlation between replicates, similarities between all samples were estimated using pair-wise Pearson correlation coefficient and plotted as a heatmap with hierarchical clustering. Analysis was then continued by enhanced Z-score ranking or using the DESeq2 R package^36^.

For the enhanced Z-score analysis, normalized read counts of replicates were collapsed to mean count values and log2 transformed. Log2 ratios were calculated between stimulated and silenced samples and normalized to enhanced Z-scores. In order to collapse to gene level, the barcode/shRNA with the strongest effect towards the positive- (for negative regulators) or negative direction (for positive regulators) was selected to represent a certain gene.

The DESeq2 package allows testing for differential expression of a gene or in this case of a barcode. Therefore, normalized read count data with all replicates for the stimulated and silenced conditions was first processed using the DESeqDataSetFromMatrix() function. Next, data were analyzed using the DESeq() function which includes the Wald test for differential expression and correction by multiple testing using the Benjamini-Hochberg method^51^.

### Orthogonal validation of selected hit shRNAs

#### Knockdown validation of selected hit shRNAs

Hit shRNAs were synthesized as two complementary oligonucleotides with AgeI and EcoRI overhangs, annealed and ligated into the pathScreener vector which has been digested with the same pair of restriction enzymes.

Target gene knockdown by hit shRNAs (see Supplementary Table S8) was validated by qRT-PCR. Therefore, primary cortical neurons were AAV infected on DIV6 with the pathScreener vector expressing individual hit shRNAs or a non-targeting control (NTC). Cultures were harvested in QIAzol cell lysis reagent (Qiagen) and total RNA was isolated with the Direct-zol RNA MiniPrep kit (Zymo). cDNA was reverse transcribed using the High-Capacity cDNA Reverse Transcription Kit (Thermo-Fisher) according to manufacturer’s instructions. Hit gene expression was quantified with intron-spanning gene specific primer pairs (see Supplementary Table S8), normalized to Rpl13a expression.

#### E-SARE luciferase reporter gene assay with individual hit shRNAs

Primary cortical neurons were AAV infected on DIV6 with the pathScreener vector expressing individual hit shRNAs or a non-targeting control (NTC). On DIV10, half of the cultures were silenced with 1 µM TTX, 100 µM D-APV (TTX cocktail) for 48 hrs. On DIV12, the remaining cultures were stimulated for 4 hours with 50 μM BIC, 100 μM 4-AP, 100 μM glycine, 1 μM strychnine (BIC cocktail). Subsequently, all cultures were lysed in passive lysis buffer (Promega). Lysate was transferred into white 96-well assay plates and measurement of luciferase bioluminescence was performed in a Mithras LB 940 Microplate Reader (Berthold Technologies) as described previously^26^.

#### GCaMP5G imaging in primary neurons

For simultaneous shRNA and GCaMP5G expression, the pathScreener vector was cut with KpnI and EcoRI to remove the sensor cassette. At this site, an insert containing the human synapsin-1 promoter (Syn1p), the GCaMP5G ORF and the SV40 poly-adenylation site was ligated^52^.

Primary cortical neurons were infected on DIV6 with AAV expressing the hit shRNA and GCaMP5G. On DIV12, cultures were stimulated with 50 μM BIC, 100 μM 4-AP, 100 μM glycine, 1 μM strychnine and GCaMP5G fluorescence was imaged on a Zeiss Observer inverted microscope. Zeiss Zen microscope software was used for image analysis. Neuronal cell bodies were selected with ring-shaped regions-of-interest to measure grey intensity values over time. Raw values were normalized to average intensity values before stimulation to obtain normalized fluorescence values (F/F_0_). Two-way ANOVA was applied for statistical analysis (GraphPad Prism).

#### Reactome and KEGG pathway analysis

Pathway analysis of the top positive regulators from the enhanced Z-score ranking was done using the Reactome database and the KEGG database^37,38^. The Reactome analysis was done using the analysis function of the Reactome Pathway Browser (http://www.reactome.org/PathwayBrowser/) and the KEGG analysis was done via the WEBGESTALT homepage (http://bioinfo.vanderbilt.edu/webgestalt/).

#### Pubmed literature search

The batch Pubmed literature search was done using the CRAN R-package RISmed for the top 150 positive regulator hit genes based on the enhanced Z-score ranking.

Search string with MeSH terms associated with synapse-to-nucleus signalling:

(“gene symbol”[Title/Abstract] OR “alternative gene symbol”[Title/Abstract] AND (neurons[MeSH Terms]) AND (“synaptic transmission”[MeSH Terms] OR “calcium signaling”[MeSH Terms] OR “MAP Kinase Signaling System”[MeSH Terms] OR “Neuronal plasticity”[MeSH Terms] OR “Long-Term Potentiation”[MeSH Terms] OR “Long-term Synaptic Depression”[MeSH Terms] OR “Neuronal Outgrowth”[MeSH Terms]OR “excitability”[Title/Abstract]).

Search string with MeSH terms associated with mental disorders:

(“gene symbol”[Title/Abstract] OR “alternative gene symbol”[Title/Abstract] AND (“neurons”[Mesh Terms]) AND (“Bipolar and Related Disorders”[MeSH Terms] OR “Mood Disorders”[MeSH Terms] OR “Neurocognitive Disorders”[MeSH Terms] OR “Schizophrenia Spectrum and Other Psychotic Disorders”[MeSH Terms]).

#### Cytoscape visualization

Protein-protein interactions were queried using the top 150 positive regulator hit genes in Bisogenet plugin from Cytoscape Apps which consolidates data from DIP, BIOGRID, HPRD, BIND, MINT and INTACT databases^53,54^. Protein identifiers were filtered for *Homo sapiens* and protein-protein interactions only with a max path length set to 2. The resulting network was further filtered to nodes with direct interaction to hit-list nodes. Edges between the non-hit-list nodes were removed to reduce the complexity of the network. Node attributes were added based on KEGG, Reactome genesets or Pubmed query results.

## Electronic supplementary material

Supplementary Table S1

Supplementary Table S2

Supplementary Table S3

Supplementary Table S4

Supplementary Table S5

Supplementary Table S6

Supplementary Table S7

Supplementary Table S8

Supplementary Information

## Data Availability

Raw sequencing data and custom computer code for data analysis will be made available upon request.
