# Supplementary material for: Pathway sensor-based functional genomics screening identifies modulators of neuronal activity
Source: Sci Rep. 2018 Dec 4;8:17597. doi: 10.1038/s41598-018-36008-9 (PMC6279925; doi:10.1038/s41598-018-36008-9)
Supplement: Supplementary file 9 — Supplementary Information [file 41598_2018_36008_MOESM9_ESM.pdf]

SUPPLEMENTARY INFORMATION to

Pathway sensor-based functional genomics screening identifies modulators of neuronal activity

Alexander Herholt[1,2], Ben Brankatschk[2], Nirmal Kannaiyan[2], Sergi Papiol[1], Sven P. Wichert[2], Michael C. Wehr[1], Moritz J. Rossner[1] *

[1] Molecular and Behavioral Neurobiology, Department of Psychiatry, Ludwig-Maximilians-University of Munich, Germany, Nussbaumstr. 7, 80336 Munich

[2] Systasy Bioscience GmbH, Adams-Lehmann-Str. 56, 80797 Munich

[*] Corresponding author: Moritz.Rossner@med.uni-muenchen.de; phone: +49 (0)89 4400 55891

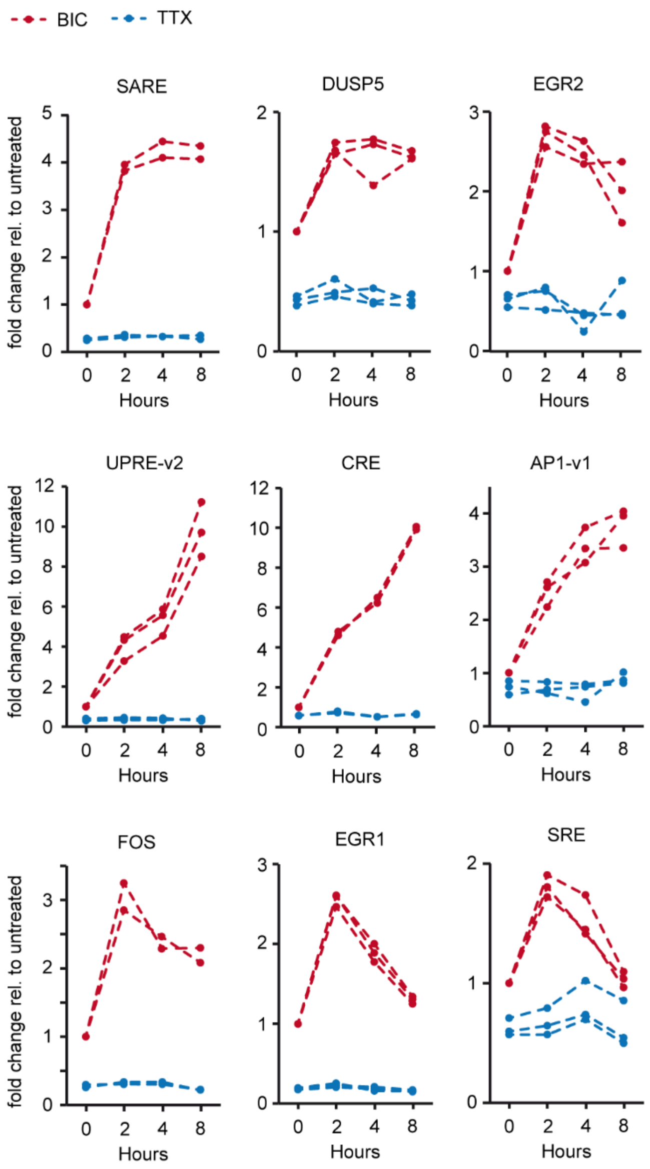

Supplementary Figure S1. Distinct kinetics of top neuronal activity sensors.

Kinetic responses of selected neuronal activity sensors from cisProfiler assays in cortical neurons upon treatment with BIC cocktail or TTX cocktail for the indicated time points (TTX treatment started 48 hr before BIC stimulation. Refers to Fig. 1). Data was normalized to the untreated control at timepoint 0 hrs. Sensors show distinct kinetic profiles which can be grouped into "sustained response" (top), "amplified response" (middle), and "transient response" (bottom) [26].

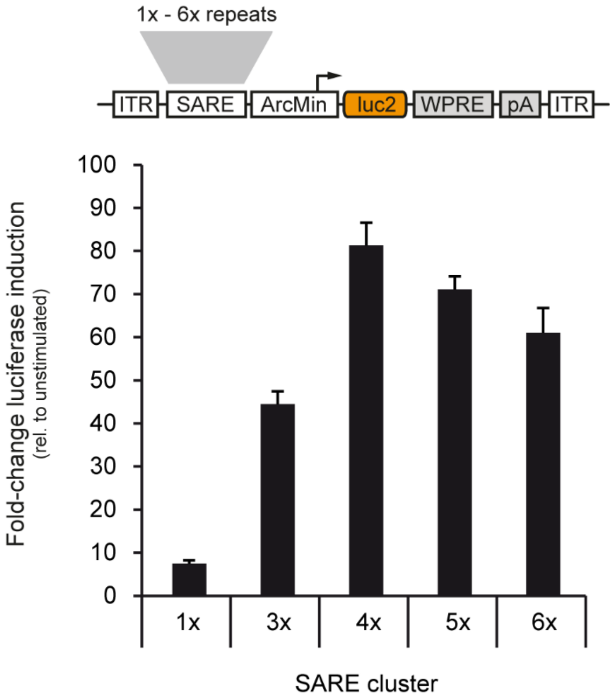

Supplementary Figure S2. SARE sensor optimization for pathScreener library.

Luciferase assay in SH-SY5Y cells, transfected with reporter plasmids harbouring different numbers of the 104 bp SARE sequence (1 and 3-6 times). Cultures were stimulated with phorbol-12-myristat-13-acetat (PMA) for 3 hrs. n = 6, ± s.d. s.d., standard deviation.

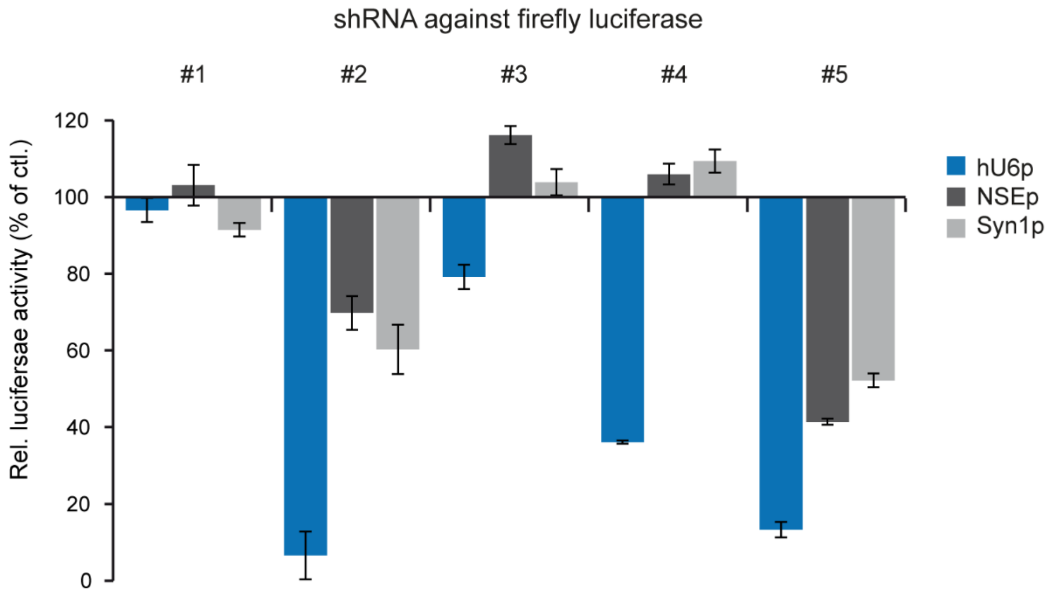

Supplementary Figure S3. Promoter impact on RNAi knockdown efficiency.

Efficiency of five different shRNAs, targeting firefly luciferase, driven by the hU6, Syn1, or NSE promoter. shRNA expression plasmids were co-transfected with a luciferase reporter plasmid into PC12 cells. hU6 promoter driven shRNAs show the strongest knockdown effect. n = 6, ± s.d. s.d., standard deviation.

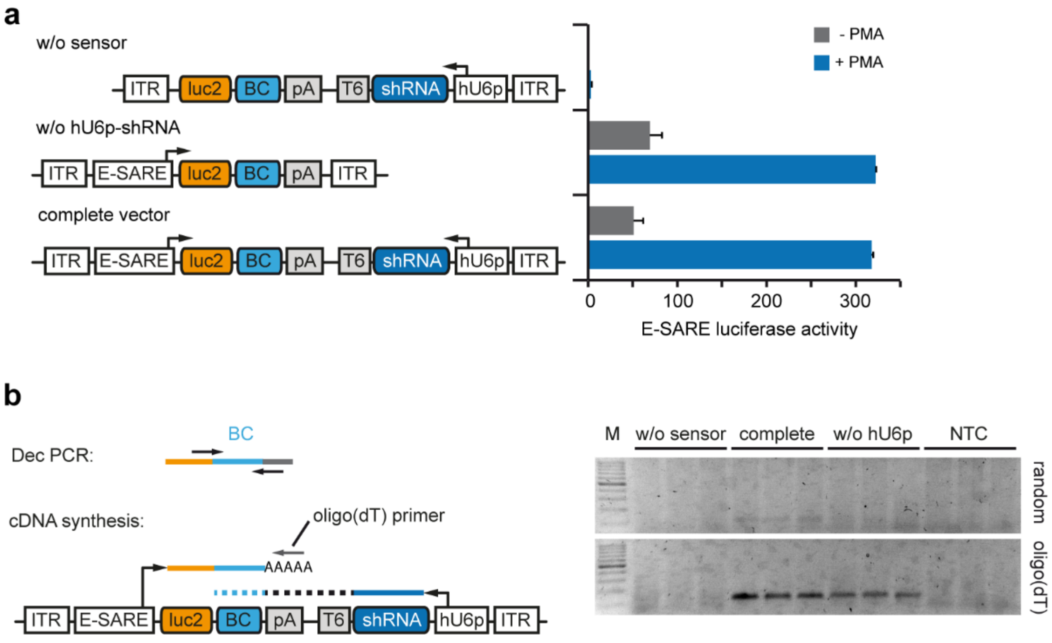

Supplementary Figure S4. Validation of the pathScreener vector.

(**a**) Validation of an unbiased sensor response in the dual-expression pathScreener vector. PC12 cells were transfected with the complete vector or a vector with a sensor deletion (w/o sensor), or an effector deletion (w/o hU6p-shRNA). Luciferase activities were measured for unstimulated and PMA-stimulated samples. n = 6, ± s.d. (**b**) Left, schematic of the vector with transcripts expressed by the sensor and by the hU6 promoter. The dashed line indicates putative DNA polymerase III run-through transcripts. Right, PC12 cells were transfected with the vectors shown in **a** and purified RNA was reverse transcribed either with random primers or oligo(dT) primers. A prominent Dec PCR barcode product is only detectable with cDNA transcribed using oligo(dT) primers. The gel image was cropped from different parts of the same gel. NTC, non-template control. s.d., standard deviation.

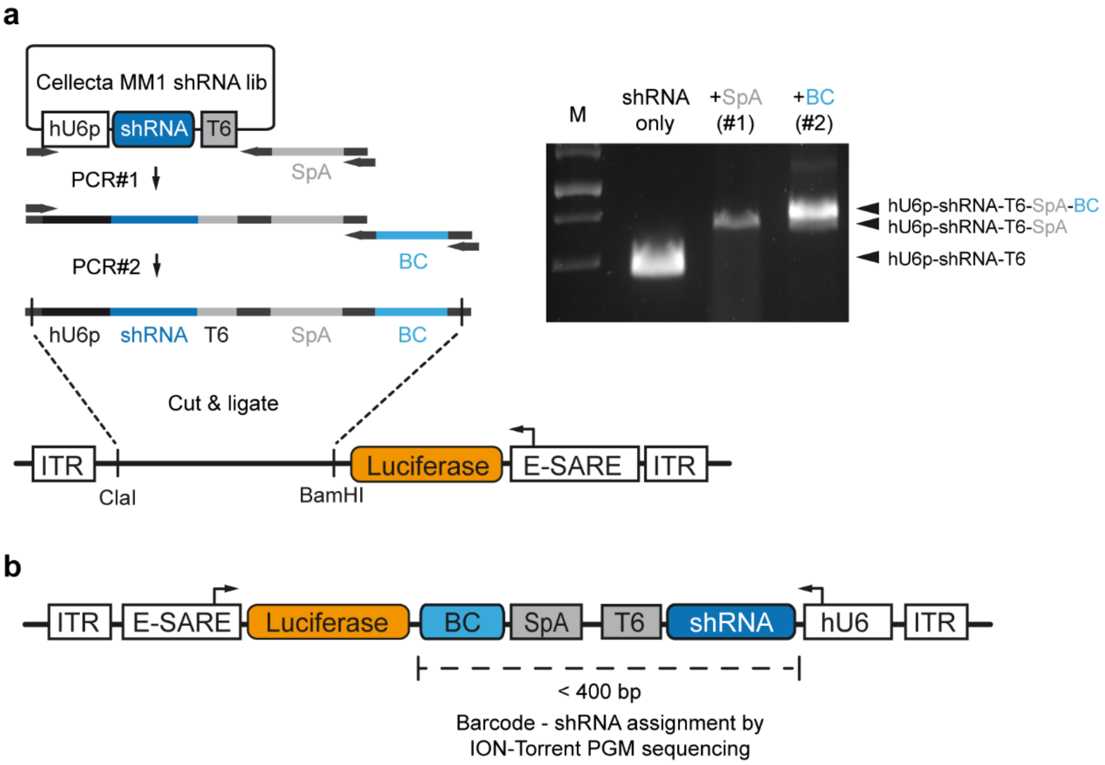

Supplementary Figure S5. pathScreener library construction.

(**a**) Cloning workflow. Left, the effector cassette was amplified by PCR and extended by the synthetic poly-A sequence (SpA). A second PCR added the random barcode (BC). The final product was digested and ligated into the sensor containing AAV pathScreener vector. Right, verification of the PCR products. (**b**) Final cloning product. The proximity of barcode and shRNA allowed the barcode-shRNA assignment by deep sequencing.

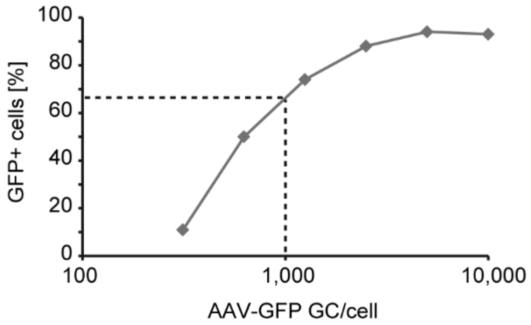

Supplementary Figure S6. AAV infection of cortical neuron cultures.

AAV infection rate for cortical neuron cultures determined using a serial dilution of a GFP expressing AAV vector. Mostly single copy infections are expected up to a viral load of 1,000 AAV genomic copies per cell (MOI 1,000). Screens A/B were performed at a MOI of 1,000 (See Fig. 2). MOI, multiplicity of infection.

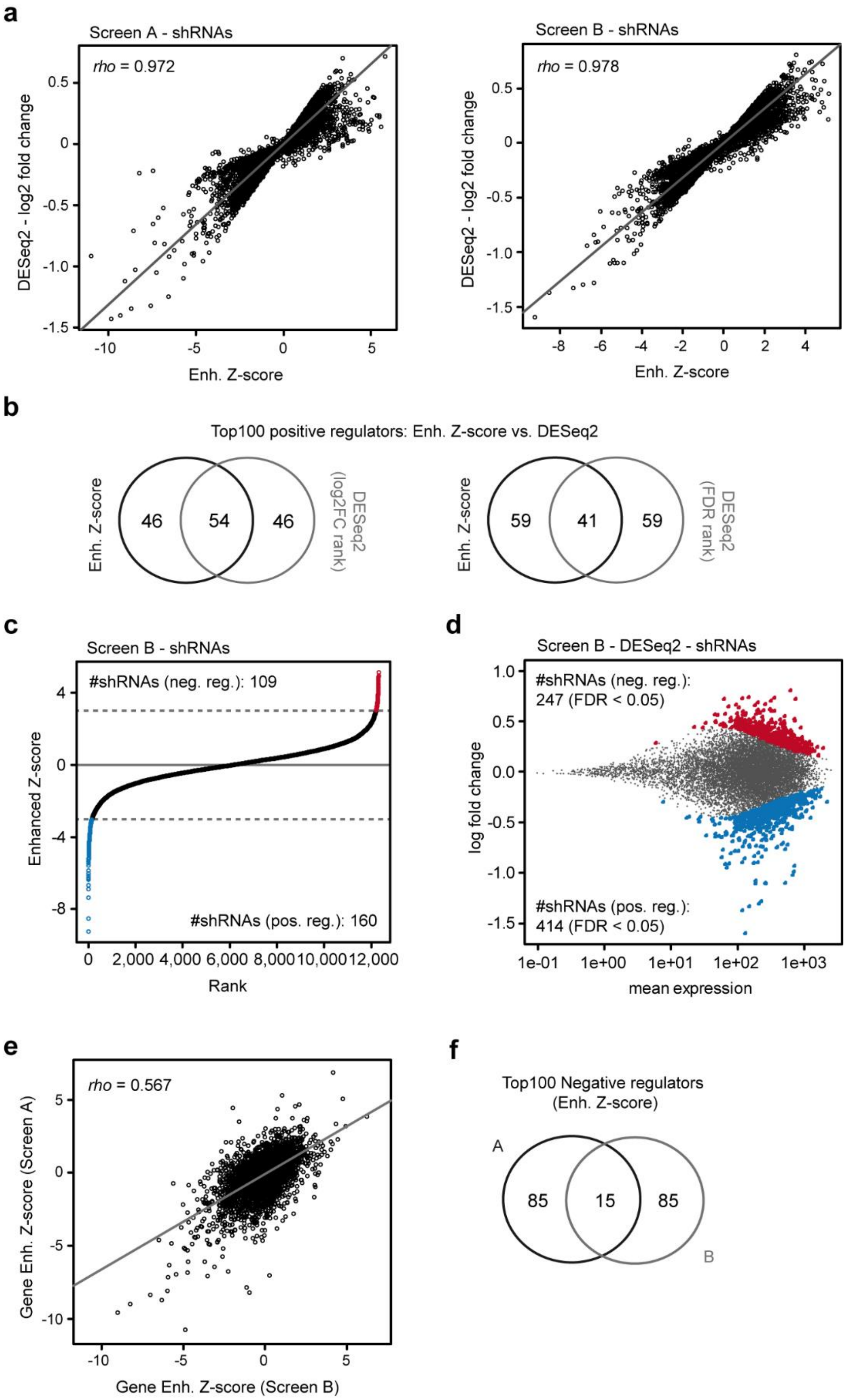

Supplementary Figure S7. Reproducibility measures of the analysis methods and the replicate screens.

(**a**) Scatterplots show the correlation between shRNA enhanced Z-scores and log2 ratios (DESeq2) for screen A (left) and B (right). Spearman-rank coefficient, *rho*. (**b**) Overlap within the top 100 positive regulators from the enhanced Z-score and DESeq2 analysis. DESeq2 hit list was either ranked by log2 fold-change (left) or by FDR (right). (**c**) Enhanced Z-score ranking for individual shRNAs from screen B. Enhanced Z-score threshold of ±3 is indicated by a dashed line. (**d**) DESeq2 analysis of screen B at shRNA level. MA-plot output comparing the sensor response for each shRNA within the library (log2 fold change: 4 hrs BIC cocktail vs. TTX cocktail; y axis) with the mean sensor expression (x axis). shRNAs with a significantly deregulated sensor expression (FDR < 0.1) are shown in red/blue. (**e**) Scatterplot compares enhanced Z-scores from screen A and B. Data has been collapsed to gene level and filtered for positive regulators. Spearman-rank coefficient, *rho*. (**f**) Overlap within the top 100 negative regulators from the enhanced Z-score analysis between screen A and B. BIC, bicuculline; TTX, tetrodotoxin; BAPTA, (1,2-bis(o-aminophenoxy)ethane-N,N,N′,N′-tetraacetic acid) ; FDR, false discovery rate.

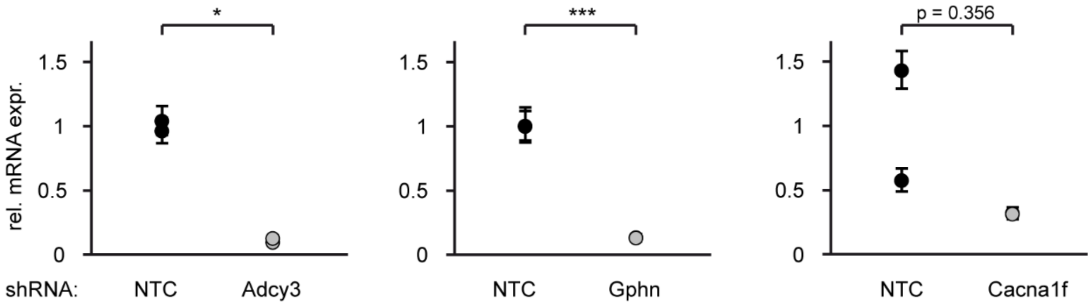

Supplementary Figure S8. Knockdown validation for *Adcy3, Gphn*, and *Cacna1f.*

Knockdown efficiencies of the top shRNAs for the hit genes *Adcy3*, *Gphn*, and *Cacna1f* (used for orthogonal validation in Fig. 3c,d). Cortical cultures were AAV infected with pathScreener vectors according to the screening paradigm (Fig. 2b). mRNA abundance relative to *Rpl13a* is shown for two biological replicates (Two-tailed T-Test). Error bars, s.d. (technical replicates). * $p < 0.05$, *** $p < 0.001$. NTC, non-targeting control vector.
